# Glycolysis in Innate Immune Cells Contributes to Autoimmunity

**DOI:** 10.3389/fimmu.2022.920029

**Published:** 2022-07-01

**Authors:** Yue Xu, Yongkang Chen, Xuan Zhang, Jie Ma, Yudong Liu, Liyan Cui, Fang Wang

**Affiliations:** ^1^ Department of Rheumatology, Beijing Hospital, National Center of Gerontology, Institute of Geriatric Medicine, Chinese Academy of Medical Sciences, Beijing, China; ^2^ Department of Laboratory Medicine, Peking University Third Hospital, Beijing, China; ^3^ Center of Biotherapy, Beijing Hospital, National Center of Gerontology; Institute of Geriatric Medicine, Chinese Academy of Medical Sciences, Beijing, China

**Keywords:** autoimmune diseases, innate immune cells, immunometabolism, glycolysis, therapeutic target

## Abstract

Autoimmune diseases (AIDs) refer to connective tissue inflammation caused by aberrant autoantibodies resulting from dysfunctional immune surveillance. Most of the current treatments for AIDs use non-selective immunosuppressive agents. Although these therapies successfully control the disease process, patients experience significant side effects, particularly an increased risk of infection. There is a great need to study the pathogenesis of AIDs to facilitate the development of selective inhibitors for inflammatory signaling to overcome the limitations of traditional therapies. Immune cells alter their predominant metabolic profile from mitochondrial respiration to glycolysis in AIDs. This metabolic reprogramming, known to occur in adaptive immune cells, i.e., B and T lymphocytes, is critical to the pathogenesis of connective tissue inflammation. At the cellular level, this metabolic switch involves multiple signaling molecules, including serine–threonine protein kinase, mammalian target of rapamycin, and phosphoinositide 3-kinase. Although glycolysis is less efficient than mitochondrial respiration in terms of ATP production, immune cells can promote disease progression by enhancing glycolysis to satisfy cellular functions. Recent studies have shown that active glycolytic metabolism may also account for the cellular physiology of innate immune cells in AIDs. However, the mechanism by which glycolysis affects innate immunity and participates in the pathogenesis of AIDs remains to be elucidated. Therefore, we reviewed the molecular mechanisms, including key enzymes, signaling pathways, and inflammatory factors, that could explain the relationship between glycolysis and the pro-inflammatory phenotype of innate immune cells such as neutrophils, macrophages, and dendritic cells. Additionally, we summarize the impact of glycolysis on the pathophysiological processes of AIDs, including systemic lupus erythematosus, rheumatoid arthritis, vasculitis, and ankylosing spondylitis, and discuss potential therapeutic targets. The discovery that immune cell metabolism characterized by glycolysis may regulate inflammation broadens the avenues for treating AIDs by modulating immune cell metabolism.

## Introduction

Autoimmune diseases (AIDs) encompass various chronic disorders involving multiple organs and have various clinical manifestations caused by connective tissue inflammation (1). Most of the current treatments for AIDs use non-selective immunosuppressive agents. Although these therapies successfully control the disease process, patients inevitably suffer from various side effects, particularly an increased risk of infection (2). Investigating the pathogenesis of AIDs is crucial for developing novel selective immunotherapies.

Immune tolerance is established during the maturation of immune cells in the bone marrow and peripheral lymphoid organs (3). Aberrant antigen presentation and differentiation of B cells into autoantibody-secreting plasma cells lead to the development of AIDs (3). Multiple signals that activate the differentiation of CD4^+^ T lymphocytes are destroyed in AIDs, leading to the breakdown of immune tolerance (4, 5). Hyperactive immune responses triggered by pathogenic autoantibodies are responsible for uncontrolled inflammation in connective tissue (6). Increasing lymphocytes accumulate in lesion locations and even form ectopic germinal centers as the disease progresses (6). However, abnormalities in cell development and function are not limited to T and B cells (7). Recently, it has been suggested that metabolic abnormalities in innate immune cells play a critical role in the pathogenesis of AIDs, such as rheumatoid arthritis (RA) and systemic lupus erythematosus (SLE) (8, 9). However, the comprehensive mechanism of innate immunity involvement in AIDs remains to be elaborated.

**Figure 1:**
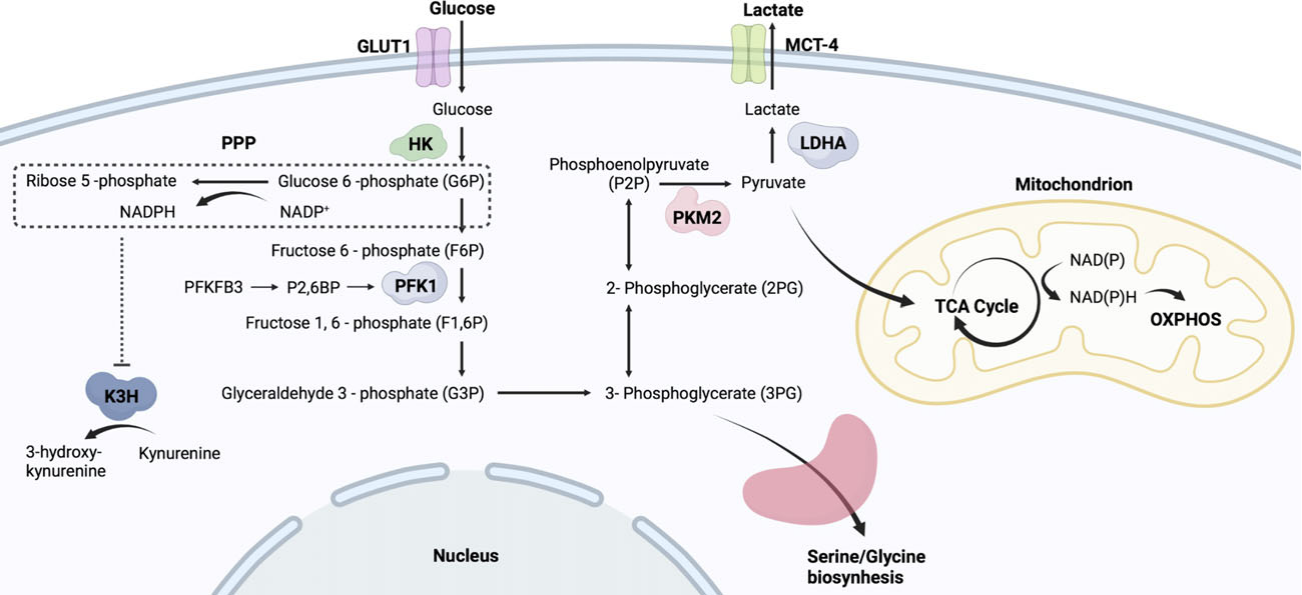
Simplified flowchart of glycolysis. Glucose entering cells is metabolized by HK to G6P, which provides a substrate for PPP. PPP generates ribose 5-phosphate and abundant NADPH. Those NADPH-dependent hydroxylases are manipulated by PPP activity, such as K3H. G6P undergoes a series of oxidative decompositions to generate 3-phosphoglycerate, providing raw materials for serine/glycine biosynthesis. PKM2 controls the final step of glycolysis and generates pyruvate. The produced pyruvate is used mainly in OXPHOS and the tricarboxylic acid TCA cycle to generate more ATP. Monocarboxylate transporter 4, MCT4; Lactic dehydrogenase A, LDHA; Phosphofructokinase-1, PFK-1; Fructose-2,6-bisphosphate, F2,6BP; Kynurenine 3-hydroxylase, K3H.

Glucose metabolism is an important metabolic pathway that provides energy to cells and consists of multiple enzymes that catalyze the conversion of glucose into metabolized products and energy in the form of ATP (10). As shown in **Figure 1**, glucose transported into the cytoplasm undergoes glycolysis, and the produced pyruvate is used mainly in oxidative phosphorylation (OXPHOS) after the tricarboxylic acid (TCA) cycle to generate more ATP. Glycolytic intermediates glucose-6-phosphate (G6P) and 3-phosphoglycerate are involved in the pentose phosphate pathway (PPP) and amino acid synthesis, respectively. In addition to providing energy, glycolytic intermediates support immune cells in reprogramming their phenotypes in response to external stimuli (11, 12). Thus, although glycolysis is less efficient than the TCA cycle or OXPHOS in producing ATP, it serves as an important metabolic pathway for activated immune cells (13). During active inflammation, immune cells use glycolysis as the major metabolic pathway to meet the demands of inflammatory activity whereas, they restore OXPHOS during the resolution of inflammation (14). Recently, it has become evident that the glycolytic switch in AIDs determines the fate of immune cells and affects the inflammatory response. More importantly, increasing metabolism has been well characterized in innate immune cells involved in AIDs (15, 16). However, the mechanism by which glycolytic activity in innate immune cells is involved in the pathogenesis of AIDs remains unclear.

In this review, we discuss the mechanisms through which glycolysis alters innate immune cells and how metabolic pathways control inflammatory responses in AIDs, thus providing insights for developing new therapeutic targets.

## Role of Glycolysis in Innate Immune Cells

**Figure 2:**
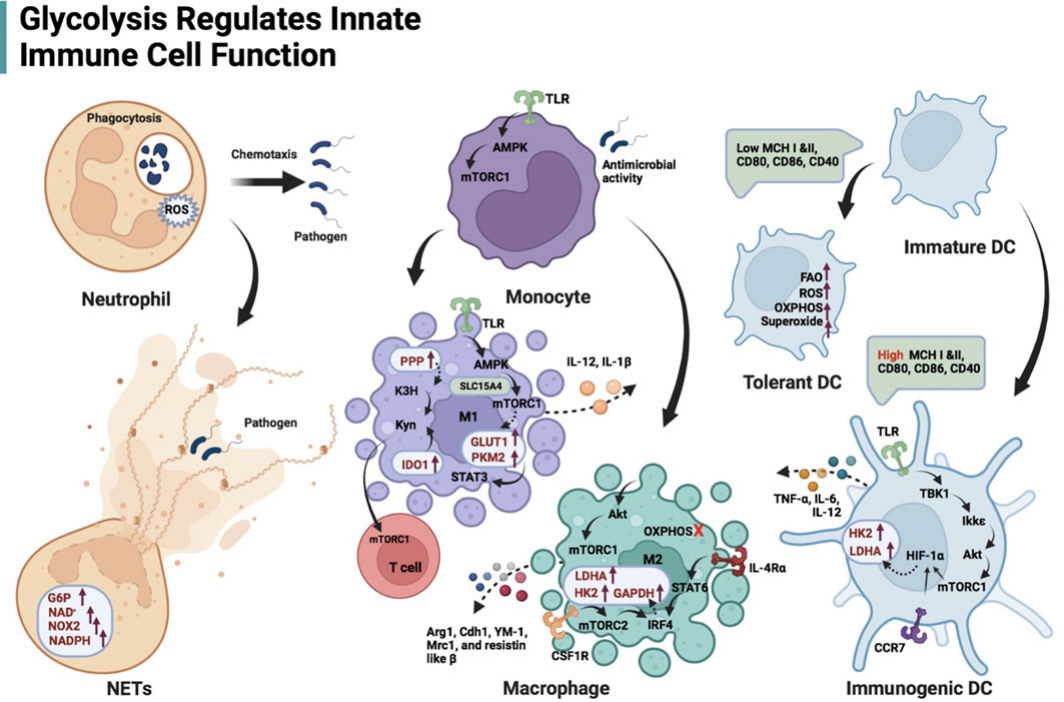
Schematic illustration of glycolysis regulating innate immune cell function. Glycolysis is the main energy production pathway for neutrophils. Impairing glycolysis and PPP can destroy neutrophil function, including chemotaxis and ROS production, even phagocytosis. NETs formation is dependent on adequate glucose flux, G6P, NOX2, and NAD^+^/NADPH. The TLR/AMPK/mTORC1 regulates glycolysis-dependent antimicrobial activity in monocytes. TLR/AMPK/mTORC1 axis is also responsible for M1-type macrophage induction, expression of glycolytic enzymes (GLUT1 and PKM2) in these cells and their IL-12 secretion. Solute carrier family 15 member A4 (SLC15A4) is likely to maintain the interaction of AMPK and mTORC1 by acting as a scaffold. PKM2 in M1 macrophages contributes to IL-1β transcription *via* STAT3 signaling. Both enhanced PPP and IDO-1 in M1 macrophages facilitate kynurenine accumulation, stimulating mTORC1 activity in T cells. Akt/mTORC1-mediated glycolysis also affects M2-like macrophage differentiation and gene profile expression (Arg1, Cdh1, YM-1, Mrc1, and resistin-like β) when OXPHOS in macrophages is inhibited. The interferon regulatory factor 4 (IRF4), which is downstream of the IL-4 receptor α/STAT6 and colony-stimulating factor 1 receptor (CSF1R)/mTORC2 signaling axis, promotes glycolysis (enhanced expression of LDHA, GAPDH and HK2) during M2 activation. DCs activated by TLRs depend on glycolysis flux to fulfill metabolic and functional requirements, including secretion of TNF-α, IL-6 and IL-12. TBK1/Ikkϵ-mediated Akt phosphorylation responds to lipopolysaccharide stimulation of TLRs on DCs. p-Akt/mTORC1 immediately promotes the transcription of HK2 and LDHA *via* HIF-1α. Cxc chemokine receptor 7 (CCR7)-mediated HIF-1α induction contributes to DC migration.

The Warburg effect is important for understanding metabolic changes that occur in innate immune cells upon activation (**Figure 2**). In resting immune cells, pyruvate can enter the TCA cycle for complete oxidation to CO_2_, generating reduced nicotinamide adenine dinucleotide and reduced flavin adenine dinucleotide (17). This process produces energy efficiently. In contrast, in an inflammatory microenvironment, pyruvate in immune cells undergoes aerobic glycolysis and regenerates nicotinamide adenine dinucleotide (NAD^+^) to rapidly meet the demands of a pro-inflammatory phenotype (17). The upregulation of glycolytic activity is caused by multiple processes, including the transfer of extracellular glucose into the cell (glucose transporter, GLUT), the breakdown of glucose (hexokinase, HK), and the conversion of glucose-6P to pyruvate (glyceraldehyde-3-phosphate dehydrogenase, GAPDH; pyruvate kinase isoenzyme M2, PKM2) (**Figure 1**) (18, 19). Besides, G6P undergoes oxidative decomposition in the PPP to generate nicotinamide adenine dinucleotide phosphate (NADPH) and ribose 5-phosphate. This metabolic branch provides the essential metabolite ribose 5-phosphate for nucleotide biosynthesis and cell proliferation (20). The abundantly produced NADPH in PPP supplies reducing power for synthetic reactions, providing antioxidant defenses for cells (20). Thus, those highly NADPH-dependent hydroxylases are manipulated by PPP activity, such as kynurenine 3-hydroxylase (21) (**Figure 1**). Immune receptors on the cell surface induce a phenotypic switch in immunometabolism. Immune receptors activate various transcription factors to induce the expression of glycolytic genes *via* kinase signaling pathways, including phosphatidylinositol 3 kinase (PI3K)/serine-threonine protein kinase (Akt), mammalian target of rapamycin (mTOR), and mitogen-activated protein kinase (MAPK) (22, 23). Hypoxia-inducible factor 1α (HIF-1α) nuclear factor kappa B (NF-kB) are major transcription factors mediating immunometabolic and inflammatory activities, inducing glucose uptake, glycolysis, and lipid synthesis (24, 25). mTOR can form different protein complexes, mTOR complexes 1 and 2 (mTORC1 and mTORC2). mTORC2 is responsible for controlling Akt activation through phosphorylation, while PI3K/Akt activates mTORC1 (17). mTORC2 enhances GLUT1 expression and aerobic glycolytic activity. mTORC1 is not only involved in the signaling of glycolysis but also promotes the synthesis of proteins and lipids (17). mTOR signaling is an important regulator of intracellular metabolic activity.

### Neutrophils

Glycolysis is the main energy production pathway for neutrophils, although glucose is also metabolized *via* the hexose monophosphate pathway. Mitochondria in neutrophils are not the main source of ATP. The mitochondria in neutrophils are primarily involved in the initiation of cell death. Therefore, the energy required for inflammatory functions and phagocytic activity originates from glycolysis in these cells (26). Phagocytosis by neutrophils involves the uptake of pathogens into plasma membrane-derived vacuoles and subsequent fusion of lysosomes with pathogen-containing phagosomes. Sodium iodoacetate, which selectively inhibits glycolysis by irreversibly inhibiting GAPDH, and 2-deoxyglucose (2-DG), which inhibits both glycolysis and PPP by competitively inhibiting G6P production, is reported to inhibit neutrophil phagocytosis (27, 28).

Neutrophils that migrate to inflammatory tissues can kill pathogens through phagocytosis, proteolytic enzymes, and reactive oxygen species (29). Neutrophils also function by releasing antimicrobial granules extracellularly (29). Antimicrobial peptides and chromatin released by neutrophils form an extracellular network that binds pathogenic microorganisms and limits the progress of infection. These extracellular fiber-structures are called neutrophil extracellular traps (NETs) (29). Scientists believe that neither NETs nor the chromatin within them originate from cell disintegration. However, mature neutrophils die within a short time after entering the circulation. The formation of NETs is likely an early event in neutrophil death (29). Various microorganisms, bacterial products, and pharmacological stimuli, such as 2-acetoxy-1-methoxypropane, induce NETs formation (30). Stimulation with 2-acetoxy-1-methoxypropane results in increased GLUT levels, glucose uptake, and glycolysis rates. However, when neutrophils are exposed to 2-acetoxy-1-methoxypropane under low glucose conditions, polymorphic nuclei are not maintained, and NETs are not formed (30). This suggests that NETs formation is strictly dependent on glucose levels. G6P, a glycolytic intermediate, enters the PPP. Nicotinamide adenine dinucleotide phosphate (NADPH) is produced during the oxidative phase of the PPP, which maintains NADPH oxidase 2 activity and reactive oxygen species (ROS) production. Both G6P and NADPH are involved in chromatin depolymerization, NADPH oxidase 2-dependent NET formation and NET release (31). NADPH appears to be the core metabolite supporting NETs. The glycolysis inhibitor 2-deoxyglucose (2-DG) affects NET formation (30). Mechanistically, after HK2 is blocked by 2-DG, G6P as the substrate for HK2 decreases immediately. Additionally, the metabolism of glucose to pyruvate in the cytoplasm generates relatively low levels of ATP and NADPH. Neutrophils use aerobic glycolysis to reduce pyruvate to lactate (which is processed by lactic dehydrogenase A, LDHA) and recycle the resultant NAD^+^ in glycolysis. Therefore, impairing LDHA also restrains the induction of NETs (32). The glucose-6-phosphate transporter/G6Pase-β complex regulates energy metabolism, glycolysis, and the PPP, in neutrophils by controlling G6P levels in the cytoplasm (31). Disturbances in glucose metabolism caused by defective glucose-6-phosphate transporter activity impair neutrophil function, including chemotaxis and ROS production (33). Moreover, NETs formation is, to some extent, dependent on glutamine and, to a lesser extent, affected by the ATP synthase inhibitor oligomycin (30).

Optic atrophy 1 (OPA1) is a mitochondrial structural protein that is essential for mitochondrial integrity and plasticity. Meanwhile, OPA1-dependent ATP is required for the formation and maintenance of NETs (34). Mechanistically, OPA1 in neutrophils can ensure the generation of NAD^+^ by maintaining the activity of electron transport complex I. Dysfunctional OPA1 reduces the NAD^+^ levels, which results in a consequent decrease in ATP from glycolysis. This suggests that glycolytic ATP plays an important role in the formation of NETs (34). Additionally, dimethyl malonate acts as a neutrophil succinate dehydrogenase inhibitor and reduces NETs release (35). These results suggest that glycolysis may be involved in NET formation through various biological mechanisms.

### Macrophages

The plasticity of macrophages enables them to switch from one phenotype to another in response to stimuli from the microenvironment (36, 37). The balance between different macrophage subtypes maintains health, while phenotype dysregulation of macrophages contributes to the development of diseases, including AIDs (38). Although the M1/M2 classification has been found insufficient to represent the diversity and complexity of macrophage subtypes as two distinct phenotypes with distinct functions, M1/M2 typing can reflect the plasticity of phenotypic transition. Interferon gamma (IFN-γ) secreted by helper T cells and bacterial lipopolysaccharide can induce macrophage differentiation to classically activated M1 (inflammatory). They produce inflammatory cytokines that are involved in pathogen elimination activities and interfere with wound healing and tissue repair (39, 40). These cytokines underlie the pathology of M1 macrophage-mediated AIDs (41). Indoleamine (2,3)-dioxygenase (IDO) is highly expressed in M1 macrophages and orchestrates tryptophan-kynurenine metabolism (42). Kynurenine stimulation alters the phenotype of human T cells *via* the eukaryotic translation initiation factor 4E binding protein 1/mTORC1 axis (43). Microenvironment-derived interleukin (IL)-21, IL-33, IL-10, and Th2-cell-derived IL-13 and IL-4 all boost macrophage polarization to the M2 type (39). This polarization appears to be a negative feedback regulation of inflammatory responses in the microenvironment, as M2-like macrophages facilitate inflammation resolution and wound healing (38) *via* secretion of the vascular endothelial growth factor and transforming growth factor-beta.

Alterations in metabolic signature support functional switching. Macrophages use OXPHOS in the resting state. M1 macrophages overexpress GLUT1 and catabolize arginine to produce nitric oxide and ROS (44). PKM2 in these cells is mobilized and phosphorylated (45), contributing to IL-1β transcription *via* signal transducer and activator of transcription (STAT) 3 signaling (46). M1 macrophages are heavily dependent on glycolysis and undergo two disruptions in the TCA cycle, resulting in an accumulation of citrate acid, succinic acid, and lactate (47). Mechanistically, 1) Downregulated isocitrate dehydrogenase caused by metabolic reprogramming inefficiently converts isocitrate to α-ketoglutarate; and 2) A large amount of itaconic acid in M1 macrophages not only limits the function of succinate dehydrogenase, resulting in substrate accumulation, but also enhances the activity of lactate dehydrogenase. As a signaling hub for toll-like receptors (TLRs), solute carrier family 15 member A4 is likely to maintain the interaction of adenosine monophosphate-activated protein kinase (AMPK) and mTORC1 by acting as a scaffold (48). Activation of TLR/AMPK/mTORC1 signaling is critical for the induction of M1-like metabolic phenotype and IL-12 secretion (48).

In contrast, M2 macrophages, which have an intact TCA cycle that provides substrates for the electron transport chain, are involved in tissue repair and wound healing and use oxidative metabolism to fuel their long-term functions (47). The inflammasome activity in M1 macrophages is regulated by NETs and ATP-binding cassette transporter A1/G1/cholesterol crystal dual signaling in tissues (49–51), followed by upregulation of several glycolytic signals such as Eno3, Aldoc, Bpgm, Pgam1, Pgam2, Pkm, and Hk3 (52). Conversely, M2 macrophages exhibit a similar level of glycolysis as unstimulated cells (53, 54). This may result from the upregulated gene expression of 6-phosphofructo-2-kinase/fructose-2,6-biphosphatase (PFKFB) 1 induced by M2-type activating signals. Phospho-fructokinase 2 encoded by PFKFB1 exhibits lower activity than that encoded by PFKFB3, thereby reducing glycolytic flux (53). Inhibiting glycolysis does not affect IL-4-induced macrophage activation without blocking OXPHOS. When OXPHOS and glycolysis were simultaneously inhibited (high doses of 2-DG, 10 mM), M2 differentiation was hindered (55). Based on the results of several recent studies, investigators suggest that glycolysis is also involved in M2 activation. Low doses of 2-DG (1 mM) can downregulate the expression of a series of M2-like gene profiles, including Arg1, Cdh1, YM-1, Mrc1, and resistin-like β through the Akt/mTORC1 signaling pathway (56–58). The IFN regulatory factor 4, which is downstream of the IL-4 receptor α/STAT6 and colony-stimulating factor 1 receptor/mTORC2 signaling axis, promotes glycolysis (enhanced expression of LDHA, GAPDH and HK2) and M2 activation (59). These results suggest that M2 activation is preferentially dependent on OXPHOS, but glycolysis is required if OXPHOS is impaired. Additional studies must elucidate the role of glycolysis in M2 activation.

Recruited by apoptotic cells, the process by which macrophages recognize, engulf, and degrade dying cells is called efferocytosis (60). Macrophages in the injured tissue exhibit exocytosis, and their metabolite load becomes equivalent to that of phagocytes (61). However, the rise in IL-10 levels observed during efferocytosis is regulated by mitochondrial β-oxidation and the electron transport chain and is not dependent on glycolysis (62). Conversely, the chemotactic behavior of macrophages in the inflammatory microenvironment relies on glycolysis, both *in vivo* and *in vitro* (63). ATP generated by glycolysis can rapidly replenish the energy required for actin synthesis and pseudopodia extension under mild hypoxia in the inflamed area (63).

Activation of myeloid-derived cells by microbial β-glucan is mediated through metabolic pathways that induce epigenetic reprogramming, also known as “trained immunity” (64). Metabolomic and transcriptomic data suggest that glutaminolysis and glycolysis are involved in β-glucan-induced immune reprogramming of monocytes. Glutamine-induced accumulation of fumarate in the TCA cycle can inhibit histone demethylase lysine-specific demethylase 5A to initiate epigenetic reprogramming in monocytes (65). This is consistent with metabolic changes in glucose mass consumption, lactate production, and inversion of the NAD^+^/NADH ratio observed in trained monocytes (52).

The Akt/mTOR/HIF-1α signaling axis regulates glycolysis in monocytes (66). Metformin activates AMPK and inhibits the antibacterial activity of monocytes that is induced by mTOR. This is also observed in the mTOR inhibitor rapamycin. Solute carrier family 15 member A4 is an amino acid/oligopeptide transporter in immune cells (67). Additionally, stimulation of the extracellular immune complex (IC) can activate HIF-1α signaling *via* the Syk/Erk/mTOR and Syk/PI3K/Akt/mTOR pathways, resulting in a switch of macrophage energy metabolism to glycolysis. Fcγ receptor IIb and aFcγ receptor are involved in the transduction of extracellular IC signals (68). This metabolic switch is observed in renal Fcγ receptor IIb-depleted macrophages associated with decreased glycolytic activity, increased mitochondrial respiratory activity, and respiratory reserves in antibody-mediated nephritis (69). Overall, these findings suggest that inhibition of glycolysis in macrophages reduces tissue inflammation, highlighting its potential as a therapeutic strategy for AIDs.

### Dendritic Cells (DCs)

As the primary antigen-presenting cells of the peripheral immune system, DCs act as a bridge between innate and adaptive immunity and are also responsible for inducing lymphocyte activation and differentiation (70). Non-activated DCs exhibit oxidative metabolism. Pathogen-associated molecular patterns bind TLRs on DCs and induce chemokines and inflammatory factors. TLRs signaling, upregulation of glycolytic activity, and increased lactate production are within the scope of Warburg physiology (71). Additionally, mitochondrial activity is progressively lost following TLR signaling. Moreover, both glucose deprivation and 2-DG blockade reverse these effects but result in decreased OXPHOS, and glucose restriction prevents DC activation, resulting in premature death of DCs (72).

This reprogramming of glucose metabolism, a switch from oxidative metabolism to glycolysis, is required for DC activation, phenotype maintenance, and migration to lymph nodes (73–75). DCs are rapidly activated by TLR signal heavily depends on a surge in glycolysis flux to fulfill metabolic and functional requirements (73), including the secretion of tumor necrosis factor-α (TNF-α), IL-6, and IL-12. Unlike conventional PI3K-dependent signaling, tumor necrosis receptor-associated factor family member associated NF-κB activator binding kinase 1 (also known as TBK1)/I-kappaB kinase ϵ-mediated Akt phosphorylation responds to lipopolysaccharide stimulation of TLRs in DCs (73). p-Akt/mTORC1 immediately promotes the transcription of HK2 and LDHA *via* HIF-1α (76). Subsequent HK2 upregulation and enrichment around the ion channels of mitochondria assist DCs to prime T cells in the microenvironment (73). Supplementing 2-DG into the DC culture mixture *in vitro* significantly weakened the ability to alter shape and remodel the cytoskeleton (74). Even the compensatory supply of ATP by mitochondrial OXPHOS could not reverse the decline in overall distance traveled and velocity (74). It has been proven that cxc chemokine receptor 7-mediated HIF-1α induction contributes to DC migration (74, 75).

Immature DCs exhibit a phenotype with lower cross-expression capacity (histocompatibility complex I and II) and lower expression of co-stimulatory molecules (CD80, CD86, and CD40) than mature DCs, which confers tolerance characteristics (i.e., tolerant DCs) in the peripheral immune system (77). Tolerant DCs exhibit significantly enhanced catabolic pathways, including OXPHOS and fatty acid oxidation, compared with the marked pro-inflammatory activity of activated DCs (also known as immunogenic DCs). Mitochondrial oxidative activity, ROS production, and superoxide production are more pronounced in tolerant DCs (78). The extracellular acidification rate (mpH/min) analysis can indicate the rate, capacity, and reserves of glycolysis. Although tolerant and immunogenic DCs exhibited similar rates of glycolysis, tolerant DCs demonstrate higher glycolytic capacity and reserves, and therefore have higher ATP reserves (78). In contrast, tolerant DCs exhibit more active fatty acid oxidation than immunogenic DCs. Inhibition of fatty acid oxidation inhibits the functions of tolerant DCs and partially restores T-cell stimulation capacity (78).

## Glycolysis in Autoimmune Diseases

The mechanism of hyperactivated glycolysis varies for different AIDs. Hallmark lesion sites represent not only differences in clinical symptoms but also differences in metabolic abnormalities. For example, expression signatures in III/IV lupus nephritis tubulointerstitium exhibit down-regulated glycolytic activity (79) while increased glucose uptake and glycolysis are observed in RA-lesioned joints (80). Additionally, glycolysis in diverse stages contributes to the development of AIDs. In the early stage of RA, naive T cells demonstrate reduced phosphofructokinase-1 activity, a deficiency of glycolysis-derived ATP, and increased cell death (81). In the late stage of RA, up-regulated GLUT1 in synovial cells of joint tissue furthers HIF-1α function (82). Therefore, blockade of hypermetabolic states and inhibition of glycolytic mediators may be therapeutically useful for AIDS. In this section, we will review the primary features of glycolysis and innate immune cells in AIDs (as shown in **Table 1**), referring to the association between glycolysis and innate immune cell function.

**Table 1:** The primary features of glycolysis and innate immune cells in AIDs.

Disease	Innate immune cell	Glycolysis activity	Mechanism	Reference
Systemic Lupus Erythematosus	Neutrophil	Down	1) The expression of GLUT-3 and GLUT-6 is decreased on the cell membranes of neutrophils in patients with SLE. 2) Deficiency of PPP leads to reduced NOX2 activity and ROS production. Reduced cellular redox capacity and oxidation of mitochondrial DNA initials NETs and neutrophil death.	(83–89)
	Monocyte/Macrophage	Up	1) Macrophages undergo a switch to glycolysis in response to IgG immune complex stimulation, which is regulated by Syk/Erk/mTOR/HIF-1α and Syk/PI3K/Akt/mTOR/HIF-1α signal. 2) Glycolysis-dependent IL-1β production in macrophages leads to neutrophil recruitment and exacerbation of lupus nephritis. 3) Hdac7 maintains PKM2 activity in macrophages via histone deacetylation. Hdac7 gene is considered a susceptibility loci for SLE. 4) PKM2 expression is highly expressed in monocytes, DCs, and B cells derived from patients with SLE, compared to those derived from healthy volunteers. A PKM2-MAPK/NF-κB-PKM2 feedback loop is activated in these cells in spontaneous lupus MRL/lpr mice and imiquimod-induced lupus mice. 5) Enhanced PPP in macrophage inhibits kynurenine 3-hydroxylase, resulting in kynurenine accumulation. Kynurenine stimulates the mTORC1 activity in human T cells.	(42, 43, 68, 90–93)
	Dendritic cell	Up	1) PKM2 expression is highly expressed in monocytes, DCs, and B cells derived from patients with SLE, compared to those derived from healthy volunteers. A PKM2-MAPK/NF-κB-PKM2 feedback loop is activated in these cells in spontaneous lupus MRL/lpr mice and imiquimod-induced lupus mice.	(90)
Rheumatoid Arthritis	Neutrophil	Up	1) NETs are triggered by the co-engagement of anti-CCP, IL-17A and TNF-α, furthering FLSs to produce IL-6 and IL-8. 2) NETs damage the cartilage matrix using elastase, MMP2, MMP8 and MMP9. 3) FLSs induced by elastase from NETs, phagocytose modified cartilage fragments, present antigens and induce autoimmune CD4+ T cells.	(94–101)
	Monocyte /Macrophage	Up	1) The SUCNR1 and accumulation of succinate induce HIF-1α, which is involved in the processing of IL-1β and arthritis exacerbation. 2) Inhibitors of TLR7 and IRAK4 interrupt the HIF-1α/c-Myc signaling. 3) Upregulated Zip8 expression causes a high production of IL-1β in monocytes/macrophages in severe arthritis via suppressing PP2A and phosphorylating mTORC1/p-S6K. 4) GSK3b expressed prevalently in the RA-injured synovium induces mitochondria in macrophages to use oxygen inefficiently to produce sufficient ATP, promoting the production of IL-1β and IL-6. 5) Hypermetabolic macrophages in RA accumulate ROS and process the transcription of IL-1β and IL-6 via PKM2/STAT3 signaling pathway. 6) Production of tumor necrosis factor-α and IL-1β are triggered by PKM2/STAT1 in macrophages. 7) The autocrine/paracrine of PKM2 promotes the differentiation of macrophages into osteoclasts, which are involved in joint destruction. Downregulation of COMMD1 protein expression by hypoxia augmented RANKL-induced expression of inflammatory and E2F1 target genes and downstream osteoclastogenesis.	(102–110)
Anti-neutrophil cytoplasmic antibody-associated vasculitis	Neutrophil	Up	1) ANCA can induce NETs formation and simultaneously promote histone citrullination, which triggers disseminated intravascular coagulation, leading to toxic damage to the endothelium.	(111)
	Monocyte/Macrophage	Up	1) Monocytes in AAV engage in glycolytic switch after ANCA stimulation. 2) Using macrophage-colony stimulating factor 1, the anti-MPO antibody stimulates monocyte differentiation into macrophages, with downregulation of IL-10, and upregulation of M1-like cytokines (IL-1β, IL-6, and IL-8).	(112–115)
Ankylosing spondylitis	Unclear	Up	1) Amino acid biosynthesis, glycolysis, glutaminolysis, fatty acid biosynthesis, and choline metabolism are significantly active in patients with AS.	(116)

Systemic Lupus Erythematosus, SLE; Glucose transporter, GLUT; Pentose phosphate pathway, PPP; Nicotinamide adenine dinucleotide phosphate, NADPH; NADPH oxidase 2, NOX2; Reactive oxygen species, ROS; Neutrophil extracellular traps, NETs; Pyruvate kinase isoenzyme M2, PKM2; Phosphatidylinositol 3 kinase, PI3K; Serine-threonine protein kinase, Akt; Mammalian target of rapamycin, mTOR; mTOR complexes 1, mTORC1; Extracellular signal-regulated kinase, Erk; Spleen associated tyrosine kinase, Syk; Hypoxia-inducible factor 1α, HIF-1α; Mitogen-activated protein kinase, MAPK; Nuclear factor kappa-light-chain-enhancer of activated B cells, NF-κB; Interleukin, IL; Signal transducer and activator of transcription, STAT; Succinate receptor 1, SUCNR1; Toll-like receptor 7, TLR7; IL-1 receptor-associated kinase 4, IRAK4; Protein phosphatase 2 phosphatase activator, PP2A; Glycogen synthase kinase 3b, GSK3b; Signal transducer and activator of transcription, STAT; Copper metabolism domain containing 1, COMMD1; Receptor activator of nuclear factor kappa-B ligand, RANKL; E2F1, E2F transcription factor 1; Matrix metalloproteinase, MMP; Anti-cyclic citrullinated peptide antibody, anti-CCP; Tumor necrosis factor-α, TNF-α; Fibroblast-like synoviocyte, FLS; Anti-neutrophil cytoplasmic antibody, ANCA; Ankylosing spondylitis, AS; Anti-neutrophil cytoplasmic antibody-associated vasculitis, AAV; Myeloperoxidase, MPO.

### SLE

Neutrophils contribute to SLE pathogenesis through multiple mechanisms, including the secretion of NETs, which are potent stimulators of type I IFN production. In SLE, neutrophil death and NETs formation are enhanced, leading to an increased debris burden associated with antinuclear autoantibodies (117–119). In healthy individuals, following uptake by macrophages, NETs are shuttled in phagosomes to lysosomes for degradation, and this process is promoted by DNase I and C1q (120). The authors showed that 62% of patients with SLE were serum positive for anti-DNase antibodies as opposed to 8% of healthy volunteers, suggesting that defects in the clearance of aberrant neutrophils by macrophages contribute to SLE pathogenesis (121). Moreover, the antimicrobial peptides and self-DNA composing the extracellular traps activate the plasmacytoid dendritic cells and autoimmune B cells *via* TLR9 engagement (122). IFN-α, a member of the type I interferon family, is majorly generated from plasmacytoid DCs in SLE (122).

The glycolytic key enzymes and metabolites are involved in the propensity to form NETs. Compared with that in neutrophils from healthy volunteers, the expression of GLUT-3 and GLUT-6 is reported to be decreased in the cell membranes of neutrophils from patients with SLE, along with a concomitant decrease in intracellular glucose concentration (83, 84). When intracellular glucose and glycolytic fluxes are declined to levels where neutrophil viability is difficult to maintain, B-cell lymphoma 2 apoptosis regulator-dependent apoptosis is activated and neutrophil numbers reduce (85). Besides, Perner et al. found that a deficiency of PPP-derived NAPDH was directly associated with reduced NADPH oxidase 2 activity and ROS production (86). Neutrophils may compensate for PPP-related ROS using mitochondrial-originated ROS. But all these changes result in reduced cellular redox capacity and oxidation of mitochondrial DNA (87). Oxidized DNA is subsequently expelled through the mechanism of NETs (87). Some scientists have revealed that neutrophils suffer from a distinct form of cell death, named NETosis, after releasing NETs (88, 89). NETosis has been indicated as an important cause of neutropenia in SLE (89). Although a recent study has revealed that ferroptosis (a novel kind of cell death related to iron overload and abnormal lipid metabolism) (123) may be the major contributor to neutropenia in SLE (124), it is undeniable that alteration of glycolytic activity is involved in the progression of this disease.

Similarly, single-cell RNA sequencing analysis of metabolism-related genes revealed decreased glycolysis and TCA cycling but increased OXPHOS in lupus nephritis renal epithelial clusters (79). The proximal tubule cells have more mitochondria than other renal epithelial cells and are therefore dependent on oxidative metabolism, which prompts the renal epithelium in lupus nephritis to develop such a metabolic switch (79). Additionally, peroxisome biogenesis signatures were markedly upregulated in proximal tubule cells, suggesting that disease progression results in altered mitochondrial and peroxisomal metabolism (79). The simultaneous administration of the mitochondrial metabolism inhibitor metformin and glycolysis inhibitor 2-DG significantly restored immune tolerance of lupus mice, dampening autoimmune inflammation. These findings indicate that immunometabolic regulation may be a practical strategy for SLE therapy (125).

Conversely, activated glycolysis regulates the functions of T cells involved in SLE pathogenesis. Glutaminase Gls1 promotes HIF-1α expression and glycolytic activity, enabling the differentiation of CD4^+^ T cells into T helper (Th) 17 cells to promote SLE disease activity (126). Aberrant tryptophan metabolites are the mediators that manipulate CD4^+^ T cells in SLE (127, 128). IDO, which is activated by IFN-γ, is primarily responsible for converting tryptophan into kynurenine and tryptamine. In an *in vitro* assay, kynurenine induces mTORC1 activity in double negative T cells but not in CD4^+^ or CD8^+^ T cells derived from human (43). Interestingly, kynurenine enhances CD4^+^ T cells to produce IFN-γ in lupus-prone mice, whereas tryptamine stimulates mTORC1 signaling and glycolytic activity in CD4^+^ T cells (129). It has been proven that gut microbiota predominantly growing in SLE contributes to this aberrant tryptophan metabolism (128, 129). IDO levels are higher in SLE patients (130) and are associated with the active phenotype in the sunny season (131). In response to inflammation, IDO is often highly expressed by antigen-presenting cells (such as macrophages and DCs) that appear specialized for rapid response (132). IL-1β alone with IDO-1 is identified as hyperactivated in autoimmune macrophages (42, 133). M1-like macrophages may be involved in generating kynurenine and modulating T-cell immunity. Besides, inhibition of kynurenine 3-hydroxylase results in decreased intracellular catabolism of kynurenine, which is driven by enhanced PPP (43). Importantly, increased PPP transcription is closely associated with CD68^+^ macrophages in the lupus kidney and is to blame for the reduced glomerular filtration rate (134). All of these show that oxidative stress in macrophages promotes kynurenine accumulation.

Recently, the glycolytic propensity in macrophages may have facilitated the inflammatory features of SLE. A study has found that human and mouse macrophages undergo a glycolytic switch in response to IgG IC stimulation, reflecting changes in macrophage metabolism in inflamed tissues *in vivo* (68). This metabolic reprogramming contributes to the production of many pro-inflammatory mediators, including IL-1β, which is further regulated by mTOR and HIF-1α. Inhibition of glycolysis or knockdown of HIF-1α attenuates IgG IC-induced macrophage activation *in vitro*, including in primary human kidney macrophage cell lines (68). Additionally, inhibition of glycolysis in a mouse model of antibody-mediated nephritis resulted in decreased renal macrophage IL-1β levels and decreased neutrophil recruitment (68).

PKM2 regulates the final step of glycolysis. PKM2 expression is reported to be higher in monocytes, DCs, and B cells derived from patients with SLE than in those derived from healthy volunteers (90). Additionally, a PKM2-MAPK/NF-κB-PKM2 feedback loop was reported in spontaneous lupus MRL/lpr mice and imiquimod-induced lupus mice (90). PKM2 inhibitors suppress proline-rich tyrosine kinase 2, preventing TLR4/TLR7/TLR9 from activating the MAPK/NF-κB pathway (90). PKM2 and lactate levels were reported to be abnormally increased in the hippocampus of MRL/lpr lupus mice (91). PKM2-β-catenin signaling leads to neuronal synapse loss by promoting microglial hyperactivation, hypersecretion of IL-6 and IL-1β, and hyperphagocytosis. *In vivo* application of AAV9-shPKM2 in a neuropsychiatric SLE mouse model was shown to delay cognitive impairment and brain damage (91). Hdac7, a histone deacetylase, prevents acetylation from limiting PKM2 activity in macrophages (92). Mouse bone marrow-specific deficient hdac7 can disrupt glycolysis-dependent inflammatory responses (92). Hdac7 is considered a susceptibility loci for SLE (93). These pieces of evidence reveal that, differing from neutrophils, controlling the glycolytic mechanism in macrophages may be a novel metabolic regulatory strategy for SLE treatment.

### RA

The metabolic microenvironment of RA exhibits different characteristics at different stages and disease sites (81). In the first stage of RA, T cells lose self-tolerance, a critical function, and facilitate autoantibody production by B cells (135). A switch in the metabolic profile within immune cells is the primary mechanism at this stage. In the second stage, autoantibodies cause metabolic alterations in synovial cells, causing uncontrolled inflammation in the joint (135). Adaptive autoimmunity initiates abnormal innate immune functions in the third stage (135).

Increasing evidence suggests metabolic feature changes in stromal and immune cells in RA (136). Recent studies have shown that the hexokinase 2 (HK2) level is elevated in Th17, DC, and fibroblast-like synoviocyte (FLS). By inhibiting glycolysis-dependent DCs activation and Th17/Treg imbalance, specific inhibition o-f HK2 (3-bromopyruvate) significantly reduced the degree of joint swelling and histological damage in SKG mice (a RA model) (137). Upregulation of HK2 is associated with hypertrophy of the synovial lining, which is involved in the bone and cartilage damage observed in RA (138). Hyperactivated HK2 promotes the proliferation and secretory function of synovial cells by mediating AMPK to activate NF-κB signaling (139). HK2 inhibitors effectively restrain the production of inflammatory factors (140). Additionally, AMPK can limit the activity of mTORC1 in immune cells. Wen et al. showed that dysfunctional AMPK induces hyperactive glycolysis in helper T cells by mediating aberrant activation of mTORC1, which facilitates the exacerbation of synovitis in RA (141). Citrate synthase levels decrease in RA synovial fluid, which suggests that the anaerobic glycolytic activity in the joint is upregulated under hypoxic conditions (142). Additionally, lactate levels in the blood samples of patients with early RA correlate with the degree of inflammation (143). Synovial fluid metabolomics in patients with RA demonstrated decreased glucose and increased lactate levels, which correlated with disease activity and markers, such as C-reactive protein (143). GLUT1 is the major glucose transporter in RA-FLSs, macrophages, and T cells. Upregulation of GLUT1 has been reported in the lining and sublining of the RA synovium (82). GLUT1 is also associated with increased glucose uptake and a hypoxic microenvironment in the joints with increased HIF-1α activity (144).

A recent study provided new insights into how glycolysis in innate immune cells contributes to RA progression. Using [^18^F]-fluoro-2-deoxy-d-glucose ([^18^F]FDG)-positron emission tomography-computed tomography tracing, Kubota et al. reported that increased glucose uptake in patients with RA correlated with disease severity and treatment response (145). Higher levels of [^18^F]FDG accumulation in swollen joints are associated with pannus rather than periarticular infiltration of inflammatory cells and are positively associated with arthritis progression (146). This observation suggests that activated macrophages contribute to the accumulation of [^18^F]FDG in the pannus, with hypoxia and cytokine stimulation promoting [^18^F]FDG uptake by macrophages.

Depending on glucose uptake and NADPH flux, NET induction is strongly correlated with joint damage and the pathogenesis of RA. The formation of anti-cyclic citrullinated peptide antibodies (anti-CCPs) and rheumatoid factor in the peripheral blood is a characteristic phenomenon of RA (94, 95). The citrullinated self-antigens recognized by anti-CCP are produced by peptidyl arginine deiminase (PAD) (94, 95). Neutrophils express aberrant levels of PAD in the synovial fluid of RA patients (94, 96). This is proved by the evidence that the PAD alone with myeloperoxidase (MPO) is located in the necrotic areas of synovial tissue (97). Aggrandized NETs are detected in the neutrophils from circulating, synovial tissue (98) and rheumatoid nodules in RA, which exhibit a correlation with anti-CCP levels (99). NETs formation is triggered by co-engagement of anti-CCP, IL-17A, and TNF-α (99). Subsequently, NETs induce further pro-inflammatory activity (including secretion of IL-6 and IL-8) in FLSs (99). Various proteins in NETs can damage the cartilage matrix and aggravate joint damage, including elastase and matrix metalloproteinase (2, 8, and 9) (100, 101, 147). Among them, elastase can promote the release of PAD2 from FLSs, which can citrullinate cartilage fragments (101). FLS phagocytose modified cartilage fragments (autoantigens), present antigens and induce autoimmune CD4^+^ T cells (101).

Both undifferentiated monocytes and macrophages in RA are in a hypermetabolic state (148). IL-1β, which is a consequence of metabolic reprogramming, seems to be a vital contributor to the pathogenesis of RA (102–108). Cinnamaldehyde inhibits the expression of the succinate receptor 1 and the accumulation of succinate, leading to restricted HIF-1α activation and ultimately limiting glycolytic activity (102). Inhibitors of TLR7 and IL-1 receptor-associated kinase 4 also interrupt HIF-1α/c-Myc signaling (103). Impairing macrophage glycolysis in RA is associated with inflammasome disassembly, decreased IL-1β production, and arthritis remission (102). The cellular membrane zinc transporter Zip8 is reported to be present at higher levels in the synovial tissue of patients with RA than in healthy volunteers (104). Bioavailable zinc in the monocyte cytoplasm is essential for protein phosphatase 2 phosphatase activator inhibition and mTORC1/p-S6K phosphorylation. Zip8 expression is associated with IL-1β production in monocytes/macrophages in severe arthritis (104). Immunostaining confirmed that glycogen synthase kinase 3b expression is prevalent in macrophages in the RA-injured synovium, and glycogen synthase kinase 3b inactivation is a metabolic switch that induces mitochondria in macrophages to use oxygen inefficiently to produce sufficient ATP (105). The resultant large amount of ATP maintains the activity of collagenase cathepsin K and promotes the production of IL-1β and IL-6 (105).

PKM2 may be a connection between glycolysis and activated macrophages in RA. PKM2 is an active enzyme involved in glycolysis in macrophages, and its dimerization depends on the intracellular ROS concentration (106). Hypermetabolic macrophages in RA accumulate ROS and activate the PKM2/STAT3 signaling pathway and transcription of IL-1β and IL-6 (106). Production of TNF-α and IL-1β is triggered by STAT1 under PKM2 stimulation in the induced RA model (Dark Agouti rats) (107). Although FLSs express higher levels of PKM2 than immune cells, obviously, FLSs extracellularly secrete a small amount. PKM2 in synovial fluid and plasma of RA patients originates from activated macrophages rather than FLSs (108). The released PKM2 facilitates macrophages to differentiate into osteoclasts involved in joint destruction (108). A hypoxic environment, active glycolysis, and massive secretion of inflammatory factors are characteristic of the macrophage-to-osteoclast transition in the RA synovium (109, 110). This process also involves a deficiency of inhibitory signals, including the copper metabolism domain-containing 1/receptor activator of the nuclear factor kappa-B ligand (RANKL) axis (110). Lack of regulation of NF-κB signaling and the transcription factor E2F1 are the primary drivers for the metabolic shift in osteoclasts (110).

### Anti-Neutrophil Cytoplasmic Antibody (ANCA)-Associated Vasculitis (AAV)

AAVs are a heterogeneous group of AIDs characterized by impaired microvasculature (149). Most of autoantibodies in most patients with ANCA-AAV are directed against autoantigens in the primary granules of neutrophils and lysosomes of monocytes, including MPO and protease 3. Patients with different autoantibodies exhibit different clinical manifestations. Patients with anti-protease 3 antibodies present with granulomatous inflammation, while patients with anti-MPO often present with sclerosis (149). Studies have reported that cross-linked ANCA Fab regions may be involved in disease progression by activating neutrophil superoxide production and inducing respiratory bursts (150, 151). Additionally, ANCA can induce NETs formation and simultaneously promote histone citrullination, which triggers disseminated intravascular coagulation, leading to toxic damage to the endothelium (111). This can be prevented by binding recombinant thrombomodulin to neutrophils *via* the macrophage 1 antigen (111). As mentioned earlier, lactate and G6P-dependent-PPP support the NET formation (31, 33, 34). These results indicate the crucial role of glycolysis in AAV.

Few studies have focused on the pathogenic role of monocytes in AAV. Recently, some evidence has found clues to how glycolysis in monocytes contributes to AAV development. It has also been suggested that ANCA F(ab)2, but not intact ANCA IgG, activates the respiratory burst of monocytes involved in AAV pathogenesis (152). The anti-MPO antibody stimulates monocyte differentiation into macrophages through macrophage-colony stimulating factor 1 (112), downregulates IL-10 secretion (113), and upregulates the secretion of several M1-like cytokines, such as IL-1β, IL-6, and IL-8 (114). Pro-inflammatory macrophages play a significant role in microvascular-targeted inflammation in AAV. O’Brien et al. reported that the metabolic transition of monocytes occurs after ANCA stimulation (115). Anti-MPO-stimulated monocytes exhibited massive uptake of glucose, increased glycolysis and OXPHOS (115). Glycolysis was also activated in anti-protease 3-stimulated monocytes, albeit for a shorter duration. Additionally, OXPHOS induces the subsequent secretion of IL-1β (115).

### Ankylosing Spondylitis (AS)

AS refers to a group of diseases called spondyloarthropathies that present with chronic back pain predominantly affecting the spine and sacroiliac joints, the diagnosis of which is often delayed (153, 154). Genetic factors (153) and environmental factors, including smoking and infection (154), are the main risk factors for AS development; however, the exact mechanisms underlying AS pathogenesis are unclear. Ou et al. established a serum metabolism-associated diagnostic panel and found 55 metabolites that were significantly different between patients with AS before and after TNF inhibitor treatment (116). Healthy volunteers and patients with AS could be differentiated using five metabolites: L-glutamic acid, arachidonic acid, L-phenylalanine, phosphocholine [18:1(9Z)/18:1(9Z)], and 1-palmitoylglycerol (area under the curve 0.998, 95% confidence interval: 0.992–1.000) (116). Pathway analysis showed that multiple pathways, including amino acid biosynthesis, glycolysis, glutaminolysis, fatty acid biosynthesis, and choline metabolism, were significantly active in patients with AS (116). This study provided new insights into AS pathogenesis; however, more mechanical studies investigating the role of immunometabolism in AS are required.

## Anti-Glycolysis Drugs That Target Innate Immune Cells

One possible use of metabolically targeted therapy is as an “adjuvant” to increase the effectiveness of co-administered biological or conventional antirheumatic drugs. Multiple pathways affect innate immune cells through different mechanisms that alter multiple aspects of the immune system and synergistically reduce the production and release of pro-inflammatory cytokines. Due to their cost-effectiveness and efficacy in metabolic reprogramming, many anti-malarial drugs, such as hydroxychloroquine and chloroquine, are also used to treat rheumatic diseases. While glycolysis plays a key role in activated FLSs, lactate levels in the FLSs of patients with RA are higher than those in the synovial fluid of patients with osteoarthritis (155). Increasing the reliance of immune cells on accelerated glycolysis makes them more vulnerable to apoptosis (155). This suggests that the biological mechanisms of metabolism-targeted drugs and their therapeutic effects deserve attention. In the section below, we review the molecular mechanisms of these drugs and their corresponding clinical applications.

### Rapamycin

mTOR is recognized as a central regulator of multiple metabolic pathways that control cell differentiation, death, and inflammatory activity. mTOR activity mediated by various kinase signals upregulates the expression of glycolytic and inflammatory genes, which in turn helps immune cells meet metabolic demands (156). Given the role of mTOR signaling in regulating inflammation, it serves as a critical link between metabolic phenotypes and AIDs.

Notably, as an mTORC1 inhibitor, N-acetylcysteine can safely reduce disease activity in patients with SLE by suppressing T-cell inflammatory factors (157). Lupus nephritis is a comorbidity in late-stage SLE. Rapamycin treatment reduced renal tissue damage and proteinuria, improved renal function, and prolonged survival in NZBW/F1 lupus-prone mice (158). Aberrant elevation in antiphospholipid antibodies is thought to be associated with liver damage in SLE (159). Rapamycin can restore liver mitochondrial function by targeting mTORC1 and effectively reducing anti-β2-glycoprotein I and anticardiolipin in SLE mice (159).

Previous evidence suggests that controlling Th17-triggered inflammation is the mechanism by which rapamycin treats SLE. KN-93 can restrain Th17 differentiation by inhibiting the calmodulin-dependent protein kinase IV/Akt/mTOR pathway and reduce disease damage in SLE mice (160, 161). A recent study found that the HIF1α-dependent glycolytic pathway in macrophages can exacerbate IgG deposition-induced lupus nephritis (68). PI3K- and Erk-mediated mTOR hyperactivation increases HIF1α transcriptional activity (68). MRL-lpr mice treated with rapamycin exhibited a decline in neutrophil recruitment, IL-1β, prostaglandin E2, and ROS secreted from macrophages (68). These results suggest a potential therapeutic application of rapamycin in targeting macrophages for SLE therapy.

In a single-arm, open-label, phase 1/2 trial of sirolimus (rapamycin) in patients with active SLE, both the British Isles Lupus Assessment Group score and the SLE Disease Activity Index decreased significantly after 12 months of treatment (162). The drug had no adverse effects on liver function or lymphocyte count. In another trial of pediatric patients with SLE, most achieved durable remission with sirolimus treatment (163). Moreover, sirolimus has greater advantages for serological reduction and glucocorticoid tapering, compared to the classic immunosuppressant tacrolimus, in treating SLE (164).

Everolimus is a 40-O-(2-hydroxyethyl) derivative of rapamycin (165). By inhibiting mTOR signaling to block IL-2 activation in T cells, everolimus prevents T-cell hyperactivation and reduces arthritis activity in patients with RA. Patients with RA treated with everolimus for 12 weeks (36.1%) had significantly higher rates of pain reduction and decreased disease activity than those treated with placebo (16.7%) (165). However, the everolimus-treated group showed more fluctuating liver function and blood lipid levels. Another mTOR inhibitor, sirolimus, relieves RA symptoms while it exhibits no impact on routine blood tests and liver and renal functions (166, 167). Additionally, an ongoing clinical trial is evaluating the efficacy of temsirolimus (a mTORC1 inhibitor; ClinicalTrials.gov identifier: NCT00076206) in patients with active RA on concomitant methotrexate therapy. These data demonstrate the potential use of rapamycin for RA treatment.

### Dimethyl fumarate

Dimethyl fumarate (DMF), an electrophilic, cell-permeable derivative of the TCA cycle metabolite fumarate, has been clinically approved as an immunomodulatory drug for treating multiple sclerosis (168). An increasing number of studies have reported that DMF mitigates the progression of AIDs by exerting effects on innate immune cells. DMF can succinylate the glycolytic enzyme GAPDH by covalently modifying cysteine residues. DMF can reportedly reduce GAPDH activity, inhibit aerobic glycolysis in bone marrow-derived cells and lymphocytes, and exert anti-inflammatory effects, both *in vitro* and *in vivo* (169). DMF reciprocally inhibits the survival, differentiation, and effector functions of Th1 and Th17 cells while promoting the development of regulatory T cells. Therefore, DMF selectively targets effector cells while sparing regulatory and naive T cells (170).

DMF induces nuclear translocation of nuclear factor E2-related factor 2 (Nrf2) and enhances NRF2 promoter activity while attenuating RANKL-mediated intracellular ROS generation (171). The latter is an osteoclast effector that inhibits RANKL-induced bone destruction (171). Activation of Nrf2 in osteoclasts/macrophages by DMF inhibits bone and joint destruction in patients with RA (171). Additionally, DMF inhibits MAPK signaling, thereby downregulating RANKL-induced expression of c-Fos, calcineurin-dependent 1, and nuclear factor of activated T cell cytoplasmic-1 (172). DMF disrupts actin ring formation by inhibiting the abovementioned signaling pathways, ultimately inhibiting the pit-forming activity of osteoclasts (172). DMF inhibits the extracellular release of high-mobility group box 1 by activating Nrf2. Simultaneously, DMF reduces the phosphorylation of p38 MAPK and extracellular signal-regulated kinase in osteoclasts (173). Additionally, DMF succinylates IL-1 receptor-associated kinase 4 in plasmacytoid DCs, preventing the binding of IL-1 receptor-associated kinase 4 to the adaptor protein MyD88 and decreasing the inflammatory cytokines IL-1, IL-18, INF-α, and TNF-α (174). Plasmacytoid DCs are activated in SLE and accumulate in the skin and produce interferons (175). DMF is potentially an important regulator of the innate immune response and may be a novel treatment strategy for SLE.

### Hexokinase Inhibitors

Hexokinase can catalyze the phosphorylation of glucose, initiating the glycolysis process. Owing to its structural similarity to glucose, 2-DG can competitively bind to HK2, inhibiting HK2 production by accumulation of phosphorylated 2-DG (176). In pre-clinical experiments, 2-DG significantly attenuated arthritis progression and reduced adaptive and innate immune cell activation in K/BxN mice (176). Cai et al. reported that inducible-nitric oxide synthase expressing M1 macrophages are significantly increased in arthritic joints and that 2-DG can effectively induce arginase 1 expressing M2 macrophages *via* the AMPK/NF-κB axis (177). Lonidamine, an inhibitor of HK1 and HK2, attenuates joint destruction in collagen-induced arthritis mice (178). The suppression of HK1 and HK2 downregulates the IL-1β and TNF-α expression and restores the anti-inflammatory activity of macrophages in the RA model. Currently, lonidamine and 2-DG are used in phase I/II trials for treating advanced cancers (179). The investigators observed only minor adverse effects, including nausea and blood glucose reduction (179).

### Metformin

Metformin was initially used in the first-line treatment of type 2 diabetes owing to its safe glucose-lowering effect (180). Increasing studies have taken advantage of the metabolism modulation property of metformin for treating various diseases, including cancer, cardiovascular disease, and AIDs (180). Metformin can modulate immunometabolism by mediating AMPK signaling. AMPK/mTOR is involved in the differentiation of CD4^+^ T cells (181). The expression of transcription factors, especially STAT, mediates mTORC1-dependent Th17 differentiation, which contributes to the development of RA (182). Additionally, metformin-activated AMPK suppresses the function of STAT3 and NF-κB, and boosts macrophages to exhibit an anti-inflammatory phenotype (183). In an Israeli cohort, patients on high-dose (2,550 mg/day) metformin had a lower risk of developing RA than those on low-dose (850 mg/day) metformin (adjusted hazard ratio of 0.62, 95% confidence interval 0.46–0.84), especially in women (184). A randomized controlled trial shows that RA patients treated with metformin for 12 weeks (80.8%) had significantly higher rates of pain reduction and decreased disease activity than those treated with placebo (54.7%) (185). No serious adverse effects were reported in these two groups.

Since glycolysis contributes to NETs formation, metformin reduced phorbol 12-myristate 13-acetate-induced NET formation *via* the AMPK/mTOR axis (186). NET-derived mitochondrial DNA induces IFNα production in pDCs, which is an important pathogenesis of SLE (186). Metformin with concomitant hydroxychloroquine in patients with mild or moderate SLE can reduce clinical flares and disease progression (186, 187).

## Summary and Prospect

Uncontrolled inflammatory bursts are a common feature of AIDs, including SLE, RA, AS, and ANCA-AAV. Various studies have demonstrated that innate immune cells adapt their metabolism to maintain or change their inflammatory phenotype. Additionally, a growing body of evidence supports the immunomodulatory properties of glycolytic metabolites in AIDs. Molecule machines, such as mTOR, AMPK, and HK2, which were initially thought to be simply regulators of cellular metabolism, are now regarded as therapeutic targets for modulating inflammatory responses. Hence, the discovery that an immune metabolism characterized by glycolysis may regulate inflammation broadens the avenues for treating AIDs. Strategies to target the abovementioned signaling molecules can be developed to modulate immune cell metabolism. Therefore, additional studies are needed to increase our understanding of the metabolic pathways in innate immune cells that are involved in the pathogenesis of AIDs, could potentially be exploited therapeutically to weaken exacerbated inflammatory responses.

## Author Contributions

LC and FW contributed to support the conception of the review. YX and YC wrote the manuscript and prepared the figures and the table. XZ, JM, and YL read, discussed, and revised the manuscript. All authors listed have made a substantial, direct, and intellectual contribution to the work and approved it for publication.

## Funding

This work was supported by the National Natural Science Foundation of China (62071011, 81788101), the Chinese Academy of Medical Science Innovation Fund for Medical Sciences (CIFMS) (2021-1-I2M-017, 2021-1-I2M-047, 2021-1-I2M-040, 2021-1-I2M-016, and 2021-1-I2M-026), the Capital’s Funds for Health Improvement and Research (2020-2-4019), and the Key Clinical Specialty Funding Project of Beijing.

## Conflict of Interest

The authors declare that the research was conducted in the absence of any commercial or financial relationships that could be construed as a potential conflict of interest.

## Publisher’s Note

All claims expressed in this article are solely those of the authors and do not necessarily represent those of their affiliated organizations, or those of the publisher, the editors and the reviewers. Any product that may be evaluated in this article, or claim that may be made by its manufacturer, is not guaranteed or endorsed by the publisher.

## Glossary

**Table:**

AID	Autoimmune disease
Erk	Extracellular signal-regulated kinase
RA	Rheumatoid arthritis
SLE	Systemic lupus erythematosus
OXPHOS	Oxidative phosphorylation
TCA	Tricarboxylic acid
G6P	glucose-6-phosphate
PPP	Pentose phosphate pathway
NAD^+^	Nicotinamide adenine dinucleotide
GLUT	Glucose transporter
HK	Hexokinase
GAPDH	Glyceraldehyde-3-phosphate dehydrogenase
PKM2	Pyruvate kinase isoenzyme M2
PI3K	Phosphatidylinositol 3 kinase
Akt	Serine-threonine protein kinase
mTOR	Mammalian target of rapamycin
MAPK	Mitogen-activated protein kinase
HIF-1α	Hypoxia-inducible factor 1α
NF-κB	Nuclera factor-kappa B
mTORC	mTOR complexe
NETs	Neutrophil extracellular traps
NADPH	Nicotinamide adenine dinucleotide phosphate
2-DG	2-deoxyglucose
ROS	Reactive oxygen species
LDHA	Lactic dehydrogenase A
OPA1	Optic atrophy 1
IL	Interleukin
STAT	Signal transducer and activator of transcription
6-biphosphatase	6-phosphofructo-2-kinase/fructose-2
PFKFB	
AMPK	AMP-activated protein kinase
TLR	Toll-like receptor
IC	Immune complex
DC	Dendritic cell
TNF-	Tumor necrosis factor-
TBK1	Tumor necrosis receptor-associated factor family member associated NF-κB activator binding kinase 1
FLS	Fibroblast-like synoviocyte
[18F]FDG	[18F]-fluoro-2-deoxy-d-glucose
anti-CCP	Anti-cyclic citrullinated peptide antibody
PAD	Peptidyl arginine deiminase
RANKL	Receptor activator of nuclear factor kappa-B ligand
ANCA	Anti-neutrophil cytoplasmic antibody
AAV	ANCA-associated vasculitis
MPO	Myeloperoxidase
M-CSF	Macrophage-colony stimulating factor 1
DMF	Dimethyl fumarate
Nrf2	Nuclear factor E2-related factor 2
MCT4	Monocarboxylate transporter 4
PFK-1	Phosphofructokinase-1
F2	Fructose-2
6BP	6-bisphosphate
